# Children perform extensive information gathering when it is not costly

**DOI:** 10.1016/j.cognition.2020.104535

**Published:** 2021-03

**Authors:** Aislinn Bowler, Johanna Habicht, Madeleine E. Moses-Payne, Niko Steinbeis, Michael Moutoussis, Tobias U. Hauser

**Affiliations:** a Max Planck UCL Centre for Computational Psychiatry and Ageing Research, London WC1B 5EH, United Kingdom; b Wellcome Centre for Human Neuroimaging, University College London, London WC1N 3BG, United Kingdom; c Centre for Brain and Cognitive Development, Birkbeck, University of London, London WC1E 7HX, United Kingdom; d UCL Institute of Cognitive Neuroscience, London WC1N 3AZ, United Kingdom; e Division of Psychology and Language Sciences, University College London, London WC1H 0AP, United Kingdom

**Keywords:** Cognitive development, Information gathering, Computational modelling

## Abstract

Humans often face decisions where little is known about the choice options. Gathering information prior to making a choice is an important strategy to improve decision making under uncertainty. This is of particular importance during childhood and adolescence, when knowledge about the world is still limited. To examine how much information youths gather, we asked 107 children (8–9 years, *N* = 30), early (12–13 years, *N* = 41) and late adolescents (16–17 years, *N* = 36) to perform an information sampling task. We find that children gather significantly more information before making a decision compared to adolescents, but only if it does not come with explicit costs. Using computational modelling, we find that this is because children have reduced subjective costs for gathering information. Our findings thus demonstrate how children overcome their limited knowledge and neurocognitive constraints by deploying excessive information gathering, a developmental feature that could inform aberrant information gathering in psychiatric disorders.

## 1 Introduction

Throughout life, people regularly encounter new situations and are forced to make decisions that they have never made before. Selecting the optimal choice option in these situations is particularly challenging because humans cannot rely on their previous experience and often have to act with only incomplete knowledge. Such situations are particularly common early in life, as children and adolescents have much more limited knowledge about the world and fewer experiences.

Humans are known to deploy a variety of strategies to solve these decisions under uncertainty. For example, we may make inference about possible outcomes by using a model of the world and relational reasoning (Tenenbaum, Kemp, Griffiths, & Goodman, 2011). We may also account for the potential information gain of a choice when valuating choice options, allowing us to make exploratory choices that are suboptimal in the short-term, but will increase performance in the long-term (Daw, O'Doherty, Dayan, Seymour, & Dolan, 2006; Dubois et al., 2020; Gershman, 2018; Wilson, Geana, White, Ludvig, & Cohen, 2014; Wu, Schulz, Speekenbrink, Nelson, & Meder, 2018). A third strategy is to postpone a decision, and to gather more decision-relevant information prior to making a choice. This information gathering allows to reduce uncertainty about choice options before committing to a choice, and thus helps to prevent making a poor choice in the first place (in part overcoming the exploration/exploitation trade-off). However, information gathering also comes with costs, as the search for information is often costly in terms of time, missed opportunities, and sometimes even financially (Bogacz, Hu, Holmes, & Cohen, 2010; Hauser et al., 2017).

In recent years, cognitive neuroscience has started to reveal the mechanisms underlying information gathering and how healthy adults solve this challenge. A key finding is that humans dynamically adjust their decision criteria depending on how long they have been sampling, and not necessarily based on explicit objective sampling costs (Bogacz, Wagenmakers, Forstmann, & Nieuwenhuis, 2010; Gesiarz, Cahill, & Sharot, 2019; Hauser, Moutoussis, Iannaccone, et al., 2017; Ma, Sanfey, & Ma, 2019; Malhotra, Leslie, Ludwig, & Bogacz, 2017; Thura, Cos, Trung, & Cisek, 2014). This behaviour is evidence for subjective sampling costs that increase as more information is gathered, rendering a long information sampling process unattractive. At an early stage, subjects only stop if there is strong evidence in favour of one option, but after extensive information gathering, participants become more lenient and are more willing to stop gathering information even if there is only slight evidence in favour of one option (Cisek, Puskas, & El-Murr, 2009; Hauser, Moutoussis, Iannaccone, et al., 2017; Malhotra et al., 2017; Moutoussis, Bentall, El-Deredy, & Dayan, 2011; Thura et al., 2014).

Being able to overcome limited information for decisions is particularly pressing during development, as children and adolescents can draw on much less prior experience to make a decision. Strikingly, children and adolescents are able to perform well on decisions under uncertainty (Gopnik, Griffiths, & Lucas, 2015; Tenenbaum et al., 2011), especially when taking their limited neurocognitive resources into account (Paus, 2005; Thompson-Schill, Ramscar, & Chrysikou, 2009; Ziegler et al., 2019). The reason for the efficient and dynamic learning in children is likely to be driven by multiple distinct cognitive features. For example, several studies have shown that children and adolescents differ in how they solve the well-known trade-off between exploration and exploitation, relying on different (often simpler) exploration strategies (Blanco & Sloutsky, 2020; Dubois et al., 2020; Meder, Wu, Schulz, & Ruggeri, 2020; Schulz, Wu, Ruggeri, & Meder, 2019). Moreover, children pro-actively direct their information search to accommodate their knowledge (Gopnik et al., 2017; Gweon, Tenenbaum, & Schulz, 2010; Meder, Nelson, Jones, & Ruggeri, 2019; Ruggeri & Lombrozo, 2014; Ruggeri, Sim, & Xu, 2017).

Relatively little is known about whether the extent of information gathering also changes during development in the transition from childhood to adolescence, which is important given the emergence of psychiatric disorders involving information gathering in youth (Fear & Healy, 1997; Kessler et al., 2005). Differently put, does development also impact how much information is gathered prior to a decision? And if so, what are the mechanisms underlying it? First evidence indeed suggests that children modulate their information gathering according to their own knowledge and external constraints (Gweon, Shafto, & Schulz, 2014), and that information gathering decreases during development (Gweon et al., 2014; Ruggeri, Lombrozo, Griffiths, & Xu, 2016; van den Bos & Hertwig, 2017). However, the computational mechanisms underlying these processes remain unclear, and little is known about whether youth are sensitive to imposed information gathering costs, and how this behaviour changes between childhood and adolescence.

In this study, we investigated how information gathering changes from late childhood (8–9 years of age) to early (12–13 years old) and late adolescence (16–17 years old). We thereby focused on the question whether the subjective costs of sampling change during this period, and whether this is dependent on the explicit costs of information gathering. We thus studied information gathering using a well-established paradigm (Clark, Robbins, Ersche, & Sahakian, 2006) that allows to capture subjective sampling costs in contexts that come with or without explicit sampling costs. Using a computational model of this information gathering task, we show that children engage in an excessive information gathering, but only if it does not incur additional explicit costs.

## 2 Materials and methods

### 2.1 Subjects

To assess age-related differences in information gathering, we recruited children and adolescents from schools across London. In total, we recruited 107 youths (62 females) in three age groups: 30 children (aged 8–9 years old (yo), mean 9.34y), 41 young adolescents (12-13yo, mean 13.13yo), and 36 older adolescents (16-17yo, mean 17.19y). We determined the sample size assuming similar, medium to large effect sizes as previous developmental studies and as our own studies with this task (e. g., Decker, Otto, Daw, & Hartley, 2016; Hauser, Moutoussis, Purg, Dayan, & Dolan, 2018; Hauser, Moutoussis, Dayan, & Dolan, 2017; Hauser, Moutoussis, Iannaccone, et al., 2017; Smid, Kool, Hauser, & Steinbeis, 2020; Somerville et al., 2017; Stenson et al., 2020). Our power simulations (10,000 simulations per group size simulation) revealed that we needed approx. 27 subjects per group to find significant group differences with a power of 80%. The groups did not differ in their age-adjusted IQ estimates (WASI-II; Wechsler, 2011) (children: 93.9 ± 13.4 (mean ± SD); young adolescents 98.5 ± 13.5; late adolescents 97.2 ± 10.3). All youths gave written informed consent, and parental consent was obtained for all participants below the age of 16. The study was approved by the UCL research ethics committee (No. 14261/001). Testing was conducted in a quiet room within the child's school and all subjects received a voucher for participating in the study (value £7). Different data from the same group of children is reported elsewhere (e.g., Dubois, Bowler, et al., 2020; Moses-Payne, Habicht, Bowler, Steinbeis, & Hauser, 2020).

### 2.2 Task

**Fig. 1:**
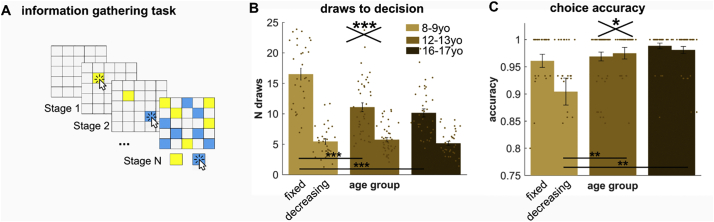
Increased information gathering in children. (A) Task procedure: Youths played an information gathering task in which they were allowed to overturn as many cards as they needed before making a decision and declaring whether the yellow or blue cards formed the majority of the 25 originally covered cards. (B) The 8–9 year-old children opened more cards than the early (12-13yo) and late (16-17yo) adolescents in the fixed condition, in which opening cards was not associated with explicit costs. There was no difference in sampling in the decreasing condition, in which their earnings decreased when opening more cards. (C) Moreover, children were slightly worse at choosing the colour that was currently more plentiful in the decreasing condition, indicating worse inference abilities. *** *p* < .001; ** *p* < .01; * *p* < .05; yo year-olds. (For interpretation of the references to colour in this figure legend, the reader is referred to the web version of this article.)

To study information gathering, we used a modified information sampling task (Clark et al., 2006;Hauser et al., 2018 ; Hauser, Moutoussis, Dayan, & Dolan, 2017 ; Hauser, Moutoussis, Iannaccone, et al., 2017). For each game (Fig. 1A), subjects were presented with 25 covered cards and they needed to decide whether the majority of the cards were either yellow or blue (colours changed on every game; cards could only ever be one of two colours on a given game). Subjects were allowed to overturn as many cards as they wished (using a computer mouse) until they felt certain enough to make their choice. Once they reached that state, they could declare which colour was in the majority by clicking on a colour button below the card deck. After each game, subjects were informed of how many points they won in the game.

We asked all subjects to play two task versions, in which we manipulated the cost of information gathering (15 games per condition). If children and adolescents are sensitive to external costs, then they should modulate their information gathering between the conditions. In a fixed-win condition, a correct choice was rewarded with +100 points, while a wrong decision was penalised by −100 points. Turning over cards did not impose any explicit costs. In the decreasing-win condition, subjects started with a potential gain of +250 points. However, each overturned card led to a reduced potential win by −10 points (e.g. after 3 draws, a potential win is +220 points). If subjects chose the wrong colour, they always lost 100 points, irrespective of how many cards they had already sampled.

To assess the extent of information gathering in each task condition, our key variable-of-interest was the average number of draws that subjects performed prior to declaring for one of the two colours. This measure reflects the need for information gathering of a subject. As a secondary outcome, we also investigated how well the subject's decision was aligned with the revealed evidence (here termed ‘accuracy’), as an indication of how precise their inference was.

### 2.3 Statistical analyses

For the behavioural analyses, we ran repeated-measures ANOVAs with a within-subject factor condition (fixed, decreasing) and a between-subject factor group (children, young adolescents, late adolescents). Significant effects were further explored using (independent samples) *t*-tests. For comparison of the model parameters, we used one-way ANOVAs with the between-subjects group factor. We report effect sizes of all analyses using Cohen's d for t-tests and partial eta-squared η^2^ for ANOVAs. Here, we present analysis results using ANOVAs, but using age as a continuous variable yielded very similar results (see Supplement).

### 2.4 Computational model

To investigate the computational mechanisms underlying age-related change in information gathering, we fitted computational models that we have previously developed for this task. We provide a summary of the model with key equations in the Supplementary Material. For a detailed discussion of the model, please see (Hauser et al., 2018; Hauser, Moutoussis, Dayan, & Dolan, 2017; Hauser, Moutoussis, Iannaccone, et al., 2017). Here we provide a description of the model and the key model parameters.

In this model, the agent makes a decision between three possible choice options: declaring for yellow, declaring for blue, or continuing with sampling (i.e. non-declaring). To make this choice, the agent assigns a value to each of these choice options. The value for declaring for blue or yellow are made based on the agent's current belief that given colour will win, multiplied with the potential win and loss amount. The agent forms this belief based on the cards that they have already opened. For example, if the agent already opened six cards and five of them were yellow, then the agent thinks it is much more likely that yellow will form the majority of cards, than if only three of the six cards were yellow.

To assign a value to the non-declaring choice option, the agent uses planning to project themselves into the future and evaluate how valuable future choices could be. It does that by thinking ahead and considering how clear a decision will be if they continue sampling another x cards, based on what the agent already knows (i.e. the cards it already opened). The quality of this planning process is thereby governed by the decision temperature parameter τ, which determines how precisely the agent plans and makes inference. A low τ thereby reflects an agent's belief to make highly rational decisions and to follow their beliefs strictly. A low τ therefore leads to a precise inference, whilst a high τ reflects a more diffuse inference process.

An additional factor that influences the value of the non-declaring option is the emergence of subjective sampling costs. Model comparison (cf Supplementary Material) showed that subjects internally represent costs for continuing sampling, even when they are not imposed by the experiment (e.g. in the fixed condition). These costs are added to the non-decision value and render it less attractive to continue sampling if the costs are high. These costs are determined by two model parameters: the scaling parameter *cs* describes how large the costs can maximally become, while parameter *p* describes when these costs start to emerge. A low *p* means that these costs incur early in the sampling process, thus promoting an early decision.

For each sample, the agent arbitrates between these three choice alternatives and hence makes predictions about what a subject should choose based on specific model parameters. To compare the model parameters between age groups, we fitted each computational model and parameter to each subject, which then provided us with the best fitting parameters for a given subject (cf Supplementary Material for details). These fitted parameters were subsequently used to make inference about the underlying development-related processes.

## 3 Results

### 3.1 Children gather more information when it's free

Three groups of children (8-9yo), early (12-13yo), and late adolescents (16-17yo) performed an information gathering task that we had extensively examined previously in adults and patients (Fig. 1A) (Hauser et al., 2018; Hauser, Moutoussis, Dayan, & Dolan, 2017; Hauser, Moutoussis, Iannaccone, et al., 2017). Youths were tasked to decide whether the majority of 25 covered cards were yellow or blue. Prior to making a decision, subjects could uncover as many cards as they wished, until they felt ‘certain enough’ with their decision. To assess the impact of external costs on information gathering, each subject played two task conditions. In the ‘fixed’ win condition, no points were deducted for gathering information prior to declaring for a colour, whereas in the ‘decreasing’ win condition the potential wins steadily decreased as a function of sampling (cf methods for more details).

To assess developmental change in information gathering, we investigated whether the groups differed in the number of cards they opened before making a final decision. We found both a significant age group effect (Fig. 1B; F(2,104) = 11.28, *p* < .001, η^2^ = 0.178) as well as a significant interaction with condition (F(2,104) = 20.43, *p* < .001, η^2^ = 0.282; main effect of condition: F(1,104) = 293.75, p < .001, η^2^ = 0.739). Subsequent *t*-tests revealed that this was driven by a significant increase in information gathering in children as compared to adolescents in the fixed (vs early adolescents: t(69) = 4.61, p < .001, d = 1.10; vs late adolescents: t(64) = 5.59, p < .001, d = 1.36), but not in the decreasing condition (vs early adolescents: t(69) = −0.50, *p* = .617, d = 0.12; vs late adolescents: t(64) = 0.54, *p* = .589, d = 0.13). No differences between the adolescent groups were observed in any of the analyses. This means that children gather significantly more information than adolescents when there is no explicit cost associated with information gathering, but show equal information gathering when sampling information is expensive. This also suggests that children deploy their information gathering in a strategic way to optimise outcomes.

### 3.2 Developmental differences in inference

Next, we investigated whether the age groups differed in other aspects of their performance. In particular, we investigated how accurate their decisions were, relative to the opened cards, i.e. whether subjects chose the colour that was more plentiful according to what they had drawn so far. We found that subjects in general performed close to ceiling but despite this, youths were more accurate in the fixed than in the decreasing condition (Fig. 1C; F(1,104) = 4.28, *p* = .041, η^2^ = 0.039). Interestingly, we found both a group main effect (F(2,104) = 8.73, p < .001, η^2^ = 0.144) and a group-by-condition interaction (F(2,104) = 3.88, *p* = .024, η^2^ = 0.069). Post-hoc *t*-tests showed that children were less accurate than adolescents and that this difference was primarily driven by differences in the decreasing condition (vs early adolescents: t(69) = −2.87, *p* = .005, d = 0.66; vs late adolescents: t(64) = −3.24, *p* = .002, d = 0.78), but much less so in the fixed condition (vs early adolescents: t(69) = −0.58, *p* = .564, d = 0.15; vs late adolescents: t(64) = −2.23, *p* = .030, d = 0.54). This means that children were less precise in their decisions, primarily in the decreasing condition where information gathering per se was already costly and where children did not differ in their information gathering prior to making a decision.

### 3.3 Excessive information gathering counteracts inference imprecision

To further understand how these behavioural effects came about and what mechanisms were underlying them, we fitted a range of different computational models to subjects' behaviour and then analysed the model parameters of the best-fitting computational model (cf Supplementary Methods; Hauser et al., 2018; Hauser, Moutoussis, Dayan, & Dolan, 2017; Hauser, Moutoussis, Iannaccone, et al., 2017). This means that the winning model was fitting the data better than the alternative models and was able to correctly predict >75% of choices (cross-validated data; chance level performance: 33%). More details can be found in Fig. S2 and the Supplementary Material.

To characterise the computational aspects that differed between the age groups, we compared the free model parameters, which can be subdivided roughly into two families. The first set of parameters (*cs*, *p*) describe the emergence of the subjective sampling costs that control the extent of information gathering prior to declaring for a colour. The other set of parameters governs the precision of the inference and decision process (ξ, τ). They determine how precisely one plans into the future, but also how this information is then used to inform decision making.

### 3.4 Altered costs drive excessive information gathering in children

One key parameter that governs when in a sampling process subjective costs start to matter (i.e. when it gets subjectively expensive to open further boxes) is parameter *p* (using separate parameters for the fixed-win and decreasing-win conditions). We have previously found that this parameter is sensitive to dissociate different groups that show differences in the number of draws (Hauser et al., 2018; Hauser, Moutoussis, Dayan, & Dolan, 2017; Hauser, Moutoussis, Iannaccone, et al., 2017).

**Fig. 2:**
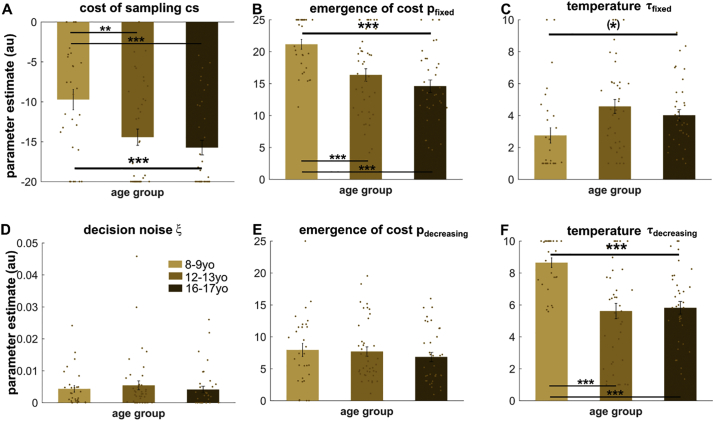
Model parameters reveal multiple distinct processes change with age. The parameters of the best-fitting model reveal that children differed from the adolescent groups across multiple model parameters. (A) Children have a lowered maximal subjective cost parameter as well as a later emergence of these costs in the fixed (B), but not in the decreasing (E) condition, which is underlying the increased information gathering behaviour in the former condition. Children also had an increased decision temperature in the decreasing (F) condition. The higher decision temperature for the decreasing condition suggests that in this more demanding setting, children perform less precise inference. A difference in decision temperature in the fixed condition did not survive multiple comparison correction (C). We found no difference in decision noise between the groups (D). *** *p* < .001; (*) uncorrected *p* < .05, which did not survive Bonferroni multiple comparison correction.

In our developmental sample, we found a significant difference between the groups in parameter *p* of the fixed condition (Fig. 2B; F(2,104) = 11.72, *p* < .001, η^2^ = 0.184). Subsequent tests revealed that this was because children had larger parameter values than young and late adolescents (vs early adolescents: t(69) = 3.63, *p* = .001, d = 0.90; vs late adolescents: t(64) = 5.13, *p* < .001, d = 1.29). There was no difference in the decreasing condition (Fig. 2E; F(2,104) = 0.49, *p* = .615, η^2^ = 0.009). In alignment with our behavioural data, this means that a critical driver underlying the increased information gathering in children is the later arising of subjective costs in the fixed condition.

Interestingly, there was also a group effect on the scaling of the costs *cs* (Fig. 2A; F(2,104) = 7.92, p = .001, η^2^ = 0.132). This was driven by a reduced maximal cost (i.e. smaller scaling parameter *cs*) in children compared to the older groups (vs early adolescent: t(69) = 2.903, *p* = .005, d = 0.69; vs late adolescents: t(64) = 3.88, p < .001, d = 0.95). This suggests that there may be multiple mechanisms at work in children, not only a delay in when these costs arise (parameter *p*), but also that sampling costs are generally perceived as less costly.

### 3.5 Reduced inference precision in children

Next, we investigated the parameters related to the precision of their inference and action. The first parameter ξ characterises a decision noise or noise floor, which accounts for decisions that were unguided by any traceable reasoning process in our model. We did not find any difference in this parameter between the three groups (Fig. 2D; F(2,104) = 0.37, *p* = .695, η^2^ = 0.007). Next, we investigated whether the inference precision parameter τ differed between age groups. This parameter not only determines how strictly you stick to the best possible option, but also governs how deterministic and precise the inference process takes place.

In the fixed condition, a difference in τ did not survive multiple comparison correction (Fig. 2C; F(2,104) = 4.49, *p* = .013 uncorrected, *p* = .078 Bonferroni corrected, η^2^ = 0.080), in line with non-significant results in the continuous age analyses (cf. Supplementary Material).

In the decreasing condition we found a significant age group effect on τ (Fig. 2F; F(2,104) = 14.76, p < .001, η^2^ = 0.221). This was driven by an increased τ in the children compared to the adolescents (vs early adolescents: t(69) = 4.88, p < .001, d = 1.23; vs late adolescents: t(64) = 5.51, p < .001, d = 1.39). This means that children make less precise inference in the decreasing condition.

## 4 Discussion

How can children make good decisions despite limited knowledge, experience, and computational resources? Here, we show that children increase the extent of their information gathering, but only if this is not costly.

Children are assumed to have limited resources and knowledge to draw from (Gopnik et al., 2015; Thompson-Schill et al., 2009), but they manage to learn and navigate the world with an astonishing sophistication. A variety of features that help children to actively learn and seek information have been identified (Tenenbaum et al., 2011). For example, children use computationally less expensive strategies and heuristics to guide their exploration (Blanco & Sloutsky, 2020; Dubois, Bowler, et al., 2020; Meder et al., 2020; Schulz et al., 2019). Moreover, children know to deploy information gathering strategies that are tailored to their existing knowledge (Gopnik et al., 2017; Gweon et al., 2010; Ruggeri et al., 2017; Ruggeri & Lombrozo, 2014). Here, we extend these findings and show that children in addition extend their information gathering, but only when it comes at no additional explicit costs.

Excessive information gathering in children may appear counter-intuitive at first, because children are believed to be generally more impulsive and avoid long deliberation (Moutoussis, Dolan, & Dayan, 2016; Olson, Hooper, Collins, & Luciana, 2007; Peters & Büchel, 2011; Steinberg et al., 2009). Moreover, lower attention span and cognitive limitations would also suggest that children may tire more quickly. However, our findings are in line with previous findings showing persisting information searches in children compared to adults (Ruggeri et al., 2016). To resolve this contradiction, it is important to acknowledge that information gathering costs are always relative to the information gain and the outcomes of the choice. The extended information gathering and reduced sampling costs (cf below) in children thus means that spending time on gathering information does not appear as costly for the children relative to the possible gains they have when making the correct decision. This could be beneficially used in educational or related settings, in which reducing the costs for a child to gather more information could benefit them and help them to learn better.

Our findings also show that children are indeed highly sensitive to external sampling costs and adjust their behaviour similar to adults when external sampling costs are imposed. In the costed condition, where gathering each piece of information incurs an explicit cost, children did not differ in their sampling behaviour. This means that children (as well as adolescents) are able to flexibly adjust their information gathering based on the external incentive structure. In the costed condition, excessive information gathering would not have been beneficial for children, because sampling more information would have led to a reduction in possible wins, thus leading to worse overall performance (cf Hauser, Moutoussis, Dayan, & Dolan, 2017; Hauser, Moutoussis, Iannaccone, et al., 2017). The condition-specific findings thus clearly show that children are sensitive to the external costs and take these into account when gathering information. This is in line with previous work, showing that children take costs into account when learning (Gweon et al., 2014). We did not observe any difference in information gathering between early and late adolescents. This could be because changes in this age range are more nuanced than between children and adolescents. Moreover, information gathering differences could be present primarily in more complex decision problems that require excessive use of cognitive maps and model-based reasoning (Schulz et al., 2019; Vaghi et al., 2020). In addition, it would be interesting to observe how information gathering changes in even younger children and to assess whether pre-school children show similar levels of information-gathering.

Our computational modelling revealed that the behavioural differences were driven by different mechanisms. Analysing the model parameters, we found that the observed increase in information gathering in children in the fixed condition is driven by a strong developmental effect on when the subjective costs arise during sampling (parameter *p*). This means that children are gathering more information because their subjective costs kick in later. We did not find any difference in that parameter in the decreasing condition, in which this parameter also captures the objective costs (i.e. the reduction in wins per sample). Interestingly, we additionally found a general scaling effect of the subjective costs with children having a lower cost scaling parameter *cs*, which affects both conditions of the task. This means that even when the costs of gathering information kick in, children still perceive these costs as less grave than adolescents. The exact nature of these intrinsic costs remain unclear and are likely to constitute a conglomerate of subjective factors, such as time costs, fatigue, or cognitive effort. Moreover, how exactly they relate to the externally imposed costs and how they are scaled relative to the potential wins and losses should be studied in more detail in future studies. Nevertheless, our findings suggest that children deploy multiple, potentially distinct, processes to increase their information gathering. Some of these processes seem to be condition-specific and primarily apply when there are no explicit costs associated with gathering information. Other processes are more general, affecting all conditions similarly.

We additionally found that the children's inference precision (parameter τ) was lower in the decreasing condition, in line with children's lower accuracy in that condition. In our model, this parameter not only governs how strongly the actual choices depend on their individual valuation, but also determines how precisely you think about the future and thus how well you can rely on the current information you have already gathered. Our finding is thus likely to align with a slow emergence of model-based reasoning (Decker et al., 2016; Smid et al., 2020; Vaghi et al., 2020) and related aspects of higher-order cognitive computations (e.g., Bolenz & Eppinger, 2020; Decker et al., 2016; Hauser, Iannaccone, Walitza, Brandeis, & Brem, 2015; Somerville et al., 2017; Tymula et al., 2012; van den Bos, Rodriguez, Schweitzer, & McClure, 2015) that require substantial resources that may not be available until later in adolescence (Thompson-Schill et al., 2009; Ziegler et al., 2019).

An inherent limitation in this cognitive resource could be overcome by gathering additional information when this comes for free, as seen in our fixed condition. This means that one can outsource the highly demanding inference process and investing in further information gathering, which simplifies the decision problem (because the difference between yellow and blue gets stronger with further sampling). This may also explain why children perform worse when they are not gathering more information, and it shows that children are strategic in their information gathering adjusting it to external circumstances. It would be interesting to further examine these mechanisms and whether the excessive information gathering in the fixed condition is linked to the children's uncertainty about the choice and planning process as, for example, captured by their confidence (cf. Moses-Payne et al., 2020; Weil et al., 2013). Moreover, it would be interesting to assess whether the effects remain if the incentive structure would be changed (e.g. sweets instead of points).

Relatively little is known about the neural mechanisms underlying information gathering and the associated sampling costs. Studies in perceptual decision making suggest that decision signals are boosted by accumulating costs that promote timely decisions (Cisek et al., 2009; Thura et al., 2014; Thura, Beauregard-Racine, Fradet, & Cisek, 2012; Thura and Cisek, 2014, Thura and Cisek, 2016). In addition, a recent pharmacological study suggests that noradrenaline may be critical for the modulation of these information costs (Hauser et al., 2018). A more detailed investigation of the neural mechanisms as well as the neurotransmitters underlying the developmental effect we observed in our study would thus be desirable.

Aberrant information gathering behaviour is also a key feature of psychiatric disorders, such as obsessive-compulsive disorder (Chamberlain et al., 2007; Dar, Rish, Hermesh, Taub, & Fux, 2000; Fear & Healy, 1997; Grassi et al., 2015; Hauser, Moutoussis, Dayan, & Dolan, 2017; Hauser, Moutoussis, Iannaccone, et al., 2017; Jacobsen, Freeman, & Salkovskis, 2012; Pélissier & O'Connor, 2002; Volans, 1976; Voon et al., 2016) or schizophrenia (Ermakova et al., 2018; Garety, Hemsley, & Wessely, 1991; Huq, Garety, & Hemsley, 1988; Jepsen et al., 2018; Moutoussis et al., 2011; So, Siu, Wong, Chan, & Garety, 2016). Interestingly, these disorders have been linked to parameters similar to the ones we found change from childhood to adolescence (Hauser, Moutoussis, Iannaccone, et al., 2017; Moutoussis et al., 2011). Whether aberrant information gathering during the transition to adolescence is linked to the emergence of these psychiatric disorders needs to be investigated in longitudinal studies.

In summary, we show that information gathering behaviour decreases between childhood and early adolescence. Using computational modelling, we show that children gather information excessively when it comes at no explicit costs. This increased information gathering may be beneficial in situations in which little is known and where learning can only take place once the choice options are better understood. These findings may help understand how different information gathering impairments arise in adolescence-related psychiatric disorders.

## Data and code availability

Data is available from the corresponding author upon reasonable request. A toolbox with the computational model is currently in preparation.

## Declaration of Competing Interest

None.

## Appendix A Supplementary data

Supplementary material Image 1
