# Supplementary material for: Children perform extensive information gathering when it is not costly
Source: Cognition. 2021 Mar;208:104535. doi: 10.1016/j.cognition.2020.104535 (PMC7871012; doi:10.1016/j.cognition.2020.104535)
Supplement: Supplementary file 1 — Supplementary material [file mmc1.pdf]

Children perform extensive information gathering when it is not costly

Bowler A, Habicht J, Moses-Payne ME, Steinbeis N, Moutoussis M & Hauser TU

Appendix A - Supplementary material

Computational modelling

To understand the neurocomputational mechanisms underlying information gathering, we used a model previously developed for this task. Here, we present its key features - a detailed description of the model can be found in Hauser et al., (2017b) and subsequent papers (Hauser et al., 2017a, 2018).

The computational model assumes that agents make inference about which colour is more likely to form the majority of cards $P(MY \mid n_y, N)$ with *MY* being a majority of yellow, given the current amount of yellow cards (n_y) out of a total of N sampled cards. *P(MY)* is calculated using the current number of cards, thereby inferring the generative probability likely to have caused this proportion of yellow cards (for details cf (Hauser et al., 2017b)). The actual generative probabilities in this task were close to 50% in 2/3 of trials (probability across all draws of one trial), and close to 70% in 1/3 trials, similar to our previous study (Hauser et al., 2018).

This belief is then used calculate the value of taking the decision to declare 'yellow' or 'blue' (Q - value), weighting both potential gains and losses by the inferred probability of them taking place

$$Q(Y|n_y, N) = R_{cor}P(MY|n_y, N) + R_{inc}P(MB|n_b, N)$$

with R_{cor} and R_{inc} being the potential wins and losses (set to +100 / -100 here; for discussion cf (Hauser et al., 2018)).

A more challenging computation is the estimation of the action value for not deciding (*Q(ND)*). This is computed as the sum of the value of the future states (*V(s')*, a weighted sum of the Q values in that state), weighted by how likely they will materialise ($P(s'|n_y,N)$; i.e. how likely will I end up in that state given the cards that I have opened so far). In addition, a cost per step *c* is incurred, which accounts for the subjective costs for sampling more information

and thus spending more effort, time, and points (in the decreasing condition) on gathering information.

$$Q(ND|n_y, N) = \sum_{s'=\begin{Bmatrix} n_y+i \\ N+1 \end{Bmatrix}}^{i=[0,1]} P(s'|n_y, N)V(s') + c$$

In accordance with our previous studies (Hauser et al., 2017b, 2017a, 2018), we found that these subjective costs did not follow the explicit costs (i.e. 0 in the fixed, -10 points in the decreasing condition; named 'objective' model), and that the costs also did not follow a linear increase ('linear' model), but rather the costs follow a non-linear form (modelled as a sigmoid) in which the costs increase during sampling (Fig. S2). The best performing model had two free parameters, a scaling parameter *cs* that determined how big the maximal costs could be, and an intercept *p*, which determined after how many samples (*n*) these costs started to escalate (also cf. Drugowitsch et al., 2012; Murphy et al., 2016).

$$c = \frac{cs}{1 + e^{-10(n-p)}}$$

Lastly, the choice policy was determined using a softmax rule with decision temperature τ and an additional epsilon greedy element (ξ) that captures choices that were not adequately captured by the model. Because our model was an exact partially observable Markov decision process estimated through by dynamic programming, the policy was not only used for choice arbitration, but also in the planning process (cf Hauser et al., 2017b for details). τ thereby is not only important in arbitrating between different choice values, but also critical in the planning stages, in which this parameter portrays the precision of the inference process. A low τ means that an agent believes to make most precise decisions, which in turn means that it primarily evaluates the choice trajectories that it considers likely. A high τ on the other hand means that planning is taking place in a less precise manner (FitzGerald et al., 2014; Friston et al., 2014; Hauser et al., 2016; Schwartenbeck et al., 2019). Model comparison revealed that

models with separate decision temperature parameters performed better than ones with shared parameters (data not shown).

$$\pi(ND|n_y,N) = \frac{e^{Q(ND|n_y,N)/\tau}}{e^{Q(Y|n_y,N)/\tau} + e^{Q(B|n_y,N)/\tau} + e^{Q(ND|n_y,N)/\tau}}(1-\xi) + \frac{\xi}{3}$$

Model fitting and comparison

To determine the best fitting model, we compared different models with different forms of cost structures to it (cf above). To compare their model fit, we use out-of-sample prediction using 5-fold cross-validation assessing the predictive likelihood. This is a measure that finds an optimal balance between complexity and accuracy using the held-out data (cf. Dubois et al., 2020). We compared the models across all subjects, which allows to determine which model is the best across all subjects. Model parameters were optimised using fmincon with multiple starting points to overcome local minima. In line with our previous studies, we found that a model with nonlinear costs outperformed all other models (Fig. S2a). We additionally looked at model selection within each group to assess whether there were fundamental differences in how subjects performed the task (as captured by the models). We found that the same model that won across all subjects ('nonlinear w scaling') also won in each age group (Table S1). However, it should also be noted that the differences between the model increased by age with only very small differences between winning and second model in the children. It should also be noted that the model performance cannot be directly compared between subjects. This is because in this information gathering task, the number and art of choices depends on the information gathering behaviour. Concretely, the more information a subject gathers, the more non-decision choices are fitted using the model. It is thus only valid to compare model performance on a within-subject basis. Lastly, we conducted a parameter recovery analysis that confirmed that all parameters could well be identified (Fig. S2b).

Analysis of linear age effects

To investigate how information gathering changes during development, we gathered data from three distinct age groups and in our main analyses, we used (repeated-measures) ANOVAs to detect differences between groups. This approach is agnostic to the exact developmental trajectories and simply investigates whether there is a difference between groups. It therefore does not constrain us to a specific developmental pattern. An alternative approach is assuming a linear change with chronological age (rather than age group), which can be analysed using linear regressions. To additionally test for these linear effects, we here report the key analyses using linear regressions using the subjects' chronological age as predictor.

Age effects on task performance

Investigating the key behavioural signature in our task – number of draws before decision – we replicate the effects showing that there is a significant linear decrease in information gathering with age for the fixed (β=-.458, $p<.001$), but not for the decreasing condition (β=-.059, $p=.545$).

In addition, we found an age effect on the accuracy in both conditions, similar to the main analysis. This was stronger in the decreasing (β=.302, $p=.002$) than in the fixed condition (β=.211, $p=.029$).

Age effects on model parameters

Next, we analysed how linear age affects the model parameters. Investigating parameter *p*, we found a significant effect in the fixed condition (β=-.404, $p<.001$), but no effect in the decreasing condition (β=-.087, $p=.375$). When analysing the subjective cost of sampling parameter *cs*, we again found a significant age effect (β=-.357, $p<001$). These findings are thus

in line with the results reported in the main analysis showing multiple age-related effects in subjective sampling costs.

We then analysed the model parameters τ and found only a significant effect in the decreasing condition (β=-.374, p<.001). This parameter in the fixed condition, however, did not reach significance (β=.170, p=.081). Lastly, we again did not observe any age-effects on the noise floor parameter ξ (β=-.021, p=.831). These findings again confirm the results of the main analysis.

Table S1. Model performance separated by group. In each group the nonlinear model that included a scaling factor performed best, supporting the main model selection performed across all subjects (Fig. S2a). Winning model in bold.

	objective	subjective linear	nonlinear	nonlinear w scaling
Children	0.682±0.083	0.726±0.091	0.735±0.112	**0.736±0.090**
Early adolescents	0.695±0.062	0.748±0.069	0.750±0.077	**0.782±0.068**
Late adolescents	0.722±0.046	0.750±0.054	0.749±0.074	**0.794±0.059**

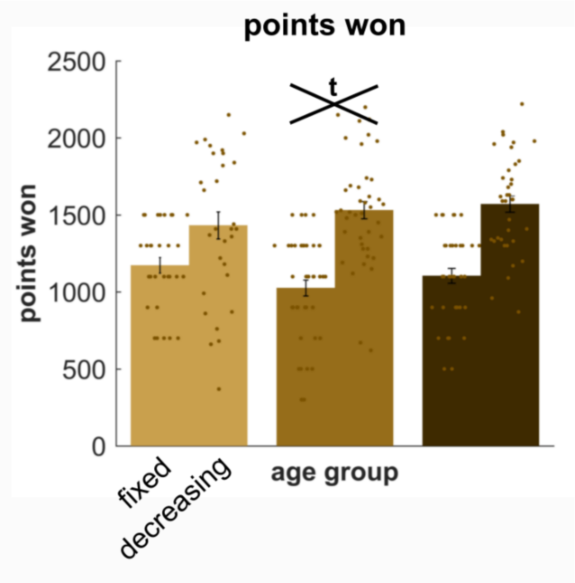

Figure S1. Children earn marginally more points in the fixed condition. We investigated overall performance as measured by the number of points subjects earned in this task. This is a rough measure of how well subjects played the task. We found – similar to our previous studies – that all subjects earned more points in the decreasing condition ($F(1,104)=83.08$, $p<.001$, $\eta 2=.44$). Whilst there was no difference between the groups ($F(2,104)=.50$, $p=.607$, $\eta 2=.01$), there was a trend for a condition-by-group interaction ($F(2,104)=2.68$, $p=.073$, $\eta 2=.049$). This was mainly driven by a slight (uncorrected) trend of children winning more points than the young adolescents in the fixed condition ($t(69)=1.96$, $p=.054$, $d=.48$), where they also opened more cards and thus made better-informed decisions. This indicates that children benefitted mildly from their increased information gathering behaviour in the fixed condition by winning marginally more points. t: $p<.10$.

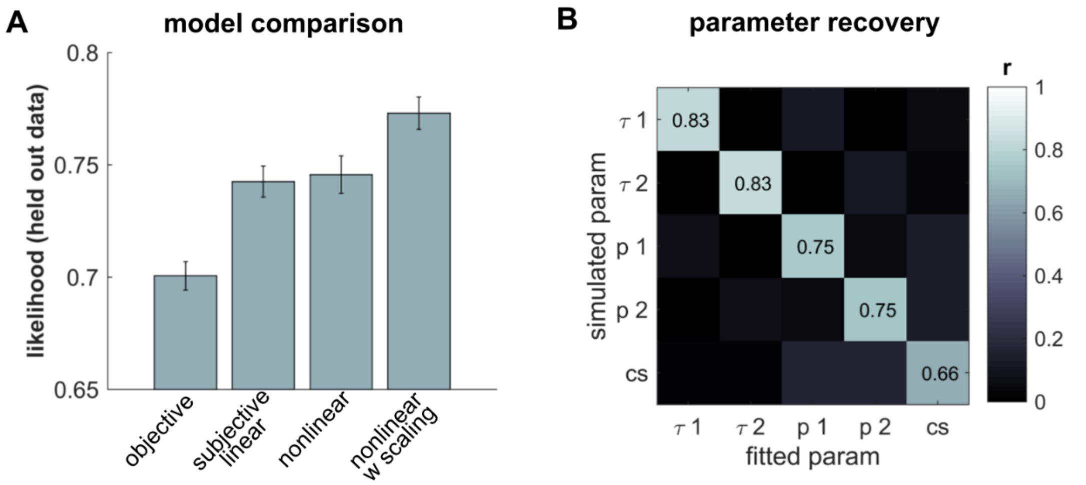

Figure S2. Model comparison. (A) Model comparison predicting cross-validated hold-out data revealed that a nonlinear increase in subjective sampling costs fits subjects' performance best. (B) Confusion matrices of the winning model shows that the parameters could be recovered using 50,000 simulated agents. 1: fixed condition, 2: decreasing condition.

References

Drugowitsch J, Moreno-Bote R, Churchland AK, Shadlen MN, Pouget A (2012) The cost of accumulating evidence in perceptual decision making. J Neurosci Off J Soc Neurosci 32:3612–3628.

Dubois M, Habicht J, Michely J, Moran R, Dolan RJ, Hauser TU (2020) Noradrenaline modulates tabula-rasa exploration. bioRxiv:2020.02.20.958025.

FitzGerald THB, Schwartenbeck P, Moutoussis M, Dolan RJ, Friston K (2014) Active Inference, Evidence Accumulation, and the Urn Task. Neural Comput:1–23.

Friston K, Schwartenbeck P, FitzGerald T, Moutoussis M, Behrens T, Dolan RJ (2014) The anatomy of choice: dopamine and decision-making. Philos Trans R Soc Lond B Biol Sci 369.

Hauser TU, Fiore VG, Moutoussis M, Dolan RJ (2016) Computational Psychiatry of ADHD: Neural Gain Impairments across Marrian Levels of Analysis. Trends Neurosci.

Hauser TU, Moutoussis M, Dayan P, Dolan RJ (2017a) Increased decision thresholds trigger extended information gathering across the compulsivity spectrum. Transl Psychiatry 7:1296.

Hauser TU, Moutoussis M, Iannaccone R, Brem S, Walitza S, Drechsler R, Dayan P, Dolan RJ (2017b) Increased decision thresholds enhance information gathering performance in juvenile Obsessive-Compulsive Disorder (OCD). PLOS Comput Biol 13:e1005440.

Hauser TU, Moutoussis M, Purg N, Dayan P, Dolan RJ (2018) Beta-blocker propranolol modulates decision urgency during sequential information gathering. J Neurosci:0192–18.

Murphy PR, Boonstra E, Nieuwenhuis S (2016) Global gain modulation generates time-dependent urgency during perceptual choice in humans. Nat Commun 7:13526.

Schwartenbeck P, Passecker J, Hauser TU, FitzGerald TH, Kronbichler M, Friston KJ (2019) Computational mechanisms of curiosity and goal-directed exploration. eLife 8.
